# Ventilatory efficiency in cardiac amyloidosis—A systematic review and meta‐analysis

**DOI:** 10.14814/phy2.70308

**Published:** 2025-05-01

**Authors:** Robin Willixhofer, Elisabetta Salvioni, Nicolò Capra, Mauro Contini, Jeness Campodonico, Piergiuseppe Agostoni

**Affiliations:** ^1^ Centro Cardiologico Monzino, IRCCS Milan Italy; ^2^ Department of Clinical Sciences and Community Health University of Milan Milan Italy; ^3^ Cento Cardiologico Monzino, L'unita di Biostatistica, IRCCS Milan Italy

**Keywords:** amyloidosis, cardiomyopathy, exercise testing, heart failure, risk prediction

## Abstract

In cardiac amyloidosis (CA) cardiopulmonary exercise testing (CPET) is underexplored. This study evaluated exercise limitations in CA using CPET, focusing on the ventilation‐to‐carbon dioxide production (VE/VCO_2_) slope and peak oxygen uptake (VO_2_). Seventeen studies involving 1505 patients were analyzed and systematically reviewed according to PRISMA reporting guidelines. Subgroup analyses assessed differences by diagnosis (ATTR vs. AL), CPET modality, and age. The cohort included 12% with AL, 80% with ATTR (23% hereditary [ATTRv], 70% wild‐type [ATTRwt], 7% unspecified), and 8% unidentified subtypes. VE/VCO_2_ slope was elevated across ATTR subgroups: 38.4 (95% CI: 36.9–40.0, *I* ^2^ = 57%) in ATTRwt and 37.9 (95% CI: 35.1–40.7, *I* ^2^ = 70%) in ATTRv. ATTR patients were older than AL patients by 9.0 years (95% CI: 0.4–17.6, *I* ^2^ = 88%) and had a higher VE/VCO_2_ slope: 2.5 (95% CI: 0.2–4.8, *I* ^2^ = 0%). CPET modality influenced peak VO_2_, which was lowest for treadmill exercise (13.7, 95% CI: 12.7–14.8, *I* ^2^ = 0%, mL/min/kg) compared to upright cycle ergometry (14.7, 95% CI: 14.3–15.1, *I* ^2^ = 33%) and semi‐recumbent cycle ergometry (14.5, 95% CI: 14.1–14.9, *I* ^2^ = 28%). A high VE/VCO_2_ slope characterizes both ATTRwt and ATTRv, while AL patients are younger with lower VE/VCO_2_ slope levels. Peak VO_2_ in ATTR patients may depend on exercise modality.

## Graphical abstract

Exercise limitations and ventilatory efficiency in cardiac amyloidosis. This illustration summarizes CPET findings in cardiac amyloidosis. Patients with transthyretin amyloidosis, both wild‐type and variant, exhibit elevated VE/VCO₂ slopes, indicating ventilatory inefficiency. Light‐chain amyloidosis patients are younger and show comparatively lower VE/VCO₂ slopes. Peak VO₂ differs by CPET modality in ATTR patients, being lowest for treadmill. These findings emphasize disease mechanisms on ventilatory efficiency and the influence of CPET modality on exercise capacity. AL, amyloid light chain amyloidosis; ATTR, transthyretin amyloidosis; ATTRv, variant transthyretin amyloidosis; ATTRwt, wild‐type transthyretin amyloidosis; CA, cardiac amyloidosis; CPET, cardiopulmonary exercise testing; VE, minute ventilation; VE/VCO₂ slope, ventilatory efficiency; VO₂, oxygen uptake.

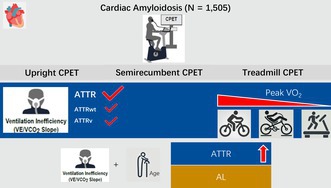

## 1 INTRODUCTION

Cardiopulmonary exercise testing (CPET) is the reference standard for the evaluation of exercise‐induced dyspnea, so that it has a particular relevance in heart failure both with reduced and with preserved ejection fraction, facilitating the differentiation of underlying pathophysiological mechanisms (American Thoracic Society & American College of Chest Physicians, 2003). It involves the measurement of respiratory gas exchange: oxygen uptake (VO_2_), carbon dioxide output (VCO_2_), and minute ventilation (VE), along with other parameters.

Various diseases exhibit distinct pathophysiological origins for dyspnea, which can be broadly categorized into three primary axes. First, cardiopulmonary limitation, including limitations of cardiac output during exercise; second, a ventilatory limitation depicted by restrictive and/or obstructive breathing patterns; and third, related to ventilatory inefficiency, being the interplay of autonomic regulations that affect dead space and ventilation/perfusion mismatch in conjunction with an abnormal ventilatory drive (Agostoni & Dumitrescu, 2019; Neder et al., 2022).

Recently, CPET gained momentum in the evaluation of patients with cardiac amyloidosis (CA), a restrictive cardiomyopathy that is caused by the infiltration of amyloid fibrils within the interstitial spaces of the myocardium (Buxbaum et al., 2022). The two most common amyloid deposits are related to monoclonal immunoglobulin light chains (AL) and transthyretin (ATTR) with the latter being further subdivided into idiopathic wild‐type (ATTRwt) and genetic variants of the disease (ATTRv) (Baker, 2022; Benson et al., 2020; Garcia‐Pavia et al., 2021; Ruberg & Berk, 2012; Tanskanen et al., 2008). Exercise testing in those patient collectives was utilized mostly to grade the exercise limitation, to evaluate the prognostic capacity, and to monitor disease specific therapy response by calculating peak VO_2_ and the VE/VCO_2_ slope (Badr Eslam et al., 2022; Cantone et al., 2023; Willixhofer et al., 2024).

The term ventilatory efficiency, represented by the VE/VCO_2_ slope, primarily describes the ability to efficiently participate in gas exchange, by the relation of VE in volume per time unit to gas exchange depicted by VCO_2_, with high values indicating a less efficient system (high ventilation in relation to low gas exchange) (Neder et al., 2022).

In fact, the evaluation by CPET of exercise limitation in CA patients, related to ventilatory inefficiency, has been suggested by us and other research groups: https://doi.org/10.1002/ehf2.15147 (Argirò et al., 2024; Bartolini et al., 2021; Hashimoto et al., 2024; Monfort et al., 2022; Nicol et al., 2021; Wernhart et al., 2024; Yunis et al., 2019). In fact, it has been recently shown that amyloid burden correlates with the VE/VCO_2_ slope, with a high burden indicating a worse ventilatory efficiency (Patel et al., 2024). Therefore, these findings highlight the importance of ventilatory efficiency assessment for the underlying pathophysiological mechanisms. Further, the underutilization of CPET in this patient cohort highlights the potential gain of clinical significance comparable to CPET in heart failure, where it serves for therapy decisions including transplantation and the evaluation of mechanical circulatory support as well as therapy success (Heidenreich et al., 2022; McDonagh et al., 2021).

Despite improved disease awareness and earlier diagnosis, CA remains classified as a rare disease (Rossi et al., 2022) with sparse evidence available in the literature for CPET. There is a need to understand if ventilatory efficiency is limited throughout the evaluated CA population, and if it varies between subgroups of the disease.

The primary aim of this systematic review and meta‐analysis is to provide a comprehensive overview of ventilatory inefficiency and exercise limitations in CA. Given the inherent heterogeneity and limited scope of available studies, this meta‐analysis serves as an exploratory effort to synthesize current evidence.

## 2 METHODS

This systematic review and meta‐analysis included a structured electronic database search for CPET in CA and a rigorous assessment of CPET variables in patients with CA overall and different forms of CA including ATTR‐CM, both ATTRwt and ATTRv, as well as AL with a consecutive subgroup analysis. Data were derived from peer‐reviewed published original investigations, therefore, no additional ethical approval was obtained. This systematic review and meta‐analysis was not registered nor had a published protocol. PRISMA reporting guidelines were followed and the corresponding checklist can be seen in Appendix S1.

### 2.1 Search strategy

The electronic database search was restricted to English‐language publication indexed MEDLINE's PubMed, which was supplemented by searching the reference lists of comprehensive reviews and all potentially relevant studies. The search strategy utilized at MEDLINE's PubMed was set by the following scheme: (“cardiopulmonary exercise testing” OR “CPET” OR “CPX” OR “exercise testing” OR “functional capacity” OR “oxygen consumption” OR “VO_2_ peak” OR “VO_2_ max” OR “ventilatory efficiency” OR “VE/VCO_2_ slope”) AND (“amyloidosis” OR “ATTR amyloidosis” OR “AL amyloidosis” OR “light chain amyloidosis” OR “transthyretin amyloidosis”) NOT animals. Search strategies were comparable and included Medical Subject Headings as appropriate. Literature search commenced on the 25th of November 2024.

### 2.2 Study selection process

Peer‐reviewed observational studies and clinical trials written in English and reporting CPET data in patients with CA were included. To ensure consistency in reported parameters, studies were required to provide VE/VCO_2_ slope data as either a primary or secondary outcome. Subgroup‐specific analyses for ATTR and AL amyloidosis were included when available.

Studies were excluded if they did not report VE/VCO_2_ slope or if they were at risk of overlapping patient cohorts. In cases of potential overlap, the study with the largest sample size and the most complete dataset from the same author or research group was included. This criterion ensured the inclusion of the most comprehensive and representative data, reducing duplication and enhancing the robustness of the analysis.

**FIGURE 1:**
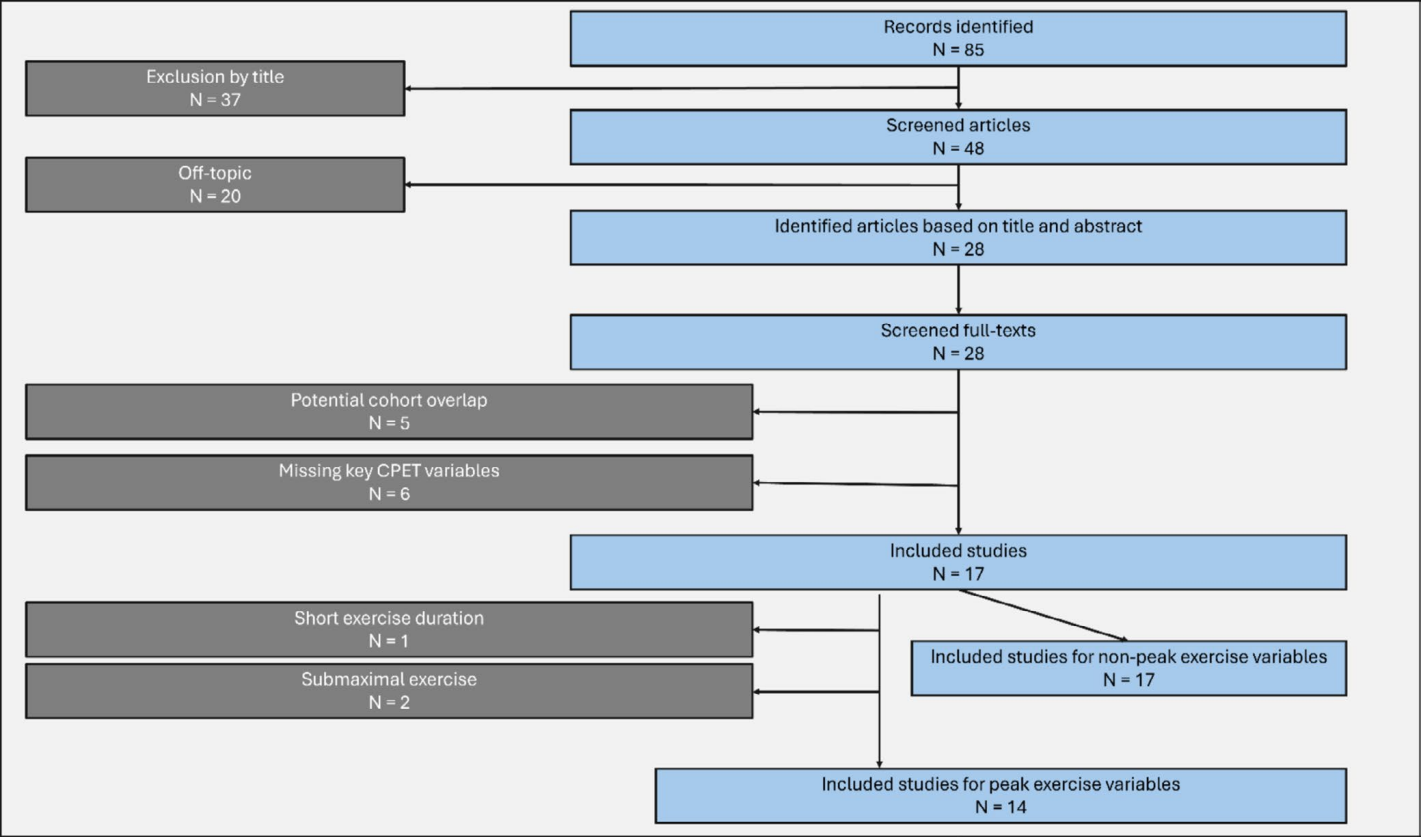
Flowchart of the study selection process. The flowchart in Figure 1 illustrates the study selection process for identifying studies relevant to cardiopulmonary exercise testing (CPET) in cardiac amyloidosis. Out of 85 initially identified records, 37 were excluded based on title screening, and 20 were excluded as off‐topic after screening articles. From 28 articles identified based on titles and abstracts, full texts were screened, resulting in the exclusion of five studies due to potential cohort overlap and six studies for missing key CPET variables. Ultimately, 17 studies were included in the final analysis. Of the 17 included studies three studies had to be excluded after subgroup analysis due to submaximal exercise and short exercise duration (8 minutes). *N*, number of studies.

The selection process was conducted by two independent reviewers who screened titles and abstracts against the inclusion and exclusion criteria. Any disagreements were resolved through discussion or consultation with a third reviewer. Notably, no discrepancies arose during the screening process. A detailed flowchart summarizing the study selection process, including reasons for exclusion, is presented in Figure 1.

### 2.3 Risk of bias

Bias was assessed using standardized tools, specifically the Newcastle‐Ottawa Scale for observational studies. While the Cochrane Risk of Bias tool is typically applied to clinical trials, no clinical trials were identified in this review, making the Newcastle‐Ottawa Scale the sole instrument used for evaluating study quality.

Two independent reviewers assessed the risk of bias, with discrepancies resolved through discussion or consultation with a third reviewer. For studies lacking sufficient information or employing unique designs that made scoring challenging, points were deducted to align with the Newcastle‐Ottawa Scale criteria and ensure a conservative bias assessment. Scoring results for all included studies are detailed in Table S1.

### 2.4 Extracted data points

The evaluation included study characteristics and patient demographics alongside CPET parameters, including the VE/VCO_2_ slope, peak VO_2_, oxygen consumption at the anaerobic threshold, and other parameters at peak exercise. All data were classified into disease subgroups where possible and applicable. Data were extracted by two independent reviewers (R.W. and E.S.) and double‐checked vice versa. If a study included follow‐up data, only baseline evaluations were included for analysis, maintaining consistency across studies.

### 2.5 Statistical analysis

In studies where data were reported as median and interquartile range without specification of the range extremes, the median was used as an estimate for the mean, and the standard deviation (SD) was estimated using Wan's method (Wan et al., 2014). For data reported as median, first quartile, and third quartile, the mean and SD were converted using the methods proposed by Luo for the mean and Wan for the SD (Luo et al., 2018; Wan et al., 2014).

Studies presenting parameters as percentages of subjects with values above a certain threshold were excluded from the analysis. If data for a higher‐level diagnosis were unavailable, they were obtained by combining the data from the subdiagnoses that compose it, such as combining ATTR and AL to obtain data for CA or combining different ATTRv mutations to obtain data for ATTRv.

This meta‐analysis was performed using the “meta” package in R software (Version 4.3.1, R Foundation for Statistical Computing, Vienna, Austria). Pooled mean estimates were calculated using the metamean function, while pooled proportions were computed with the metaprop function. The metacont function was employed to estimate mean differences for continuous variables to compare the ATTR diagnosis with the AL diagnosis and to compare ATTRwt with ATTRv. A random‐effects model with the inverse variance method was applied to determine each study's weight and aggregate the mean, proportion, and mean difference estimates.

Heterogeneity among the studies was classified as low (0 < *I* ^2^ ≤ 25%), moderate (26% < *I* ^2^ ≤ 50%), or high (*I* ^2^ > 50%). Cochran's Q test was used to assess the presence of heterogeneity across studies with *p* values of <0.05 showing statistical significance for heterogeneity. Subgroup analyses were performed to assess whether the type of diagnosis and certain characteristics of the studies (age or method of exercise) could identify different values of CPET parameters. Pooled results are presented with their respective 95% confidence intervals (95% CI).

## 3 RESULTS

The structured literature search yielded 85 results, of which 37 were excluded based on title relevance. Abstracts of the remaining 48 articles were subsequently screened. Of those, 20 were excluded to be off‐topic and after the additional exclusion of nine papers (Figure 1) the included and pooled results are derived from the 17 included studies that can be seen in Table 1. We have not excluded any study based on the quality assessment analysis via the Newcastle‐Ottawa Scale, where studies obtained a median level of 7 (Interquartile range: 6–7). Of all studies, the identification of subgroups was not clearly assessable due to differences in reporting and combining of subgroups in all cases, leading to discrepancies in the absolute numbers in assessed subgroups. However, the identifiable diagnoses yielded a total of 1505 patients with CA, which were subclassified into 181 (12%) patients with AL and 1205 (80%) with ATTR‐CM and a remaining 119 (8%) patients without differentiation in the studies. Of 1205 patients with ATTR‐CM, we identified 281 (23%) with ATTRv and 839 (70%) with ATTRwt; 85 (7%) patients were not classifiable.

**TABLE 1:** Study characteristics.

Study	Year	Aim	Study design	N	Diagnosis	CPET assessment	VE/VCO_2_ slope assesment	Conclusions	Reported limitations
Argiro et al.	2024	Evaluation of progressive changes of CPET parameters in patients with ATTR‐CM	Prospective, single center	55	ATTR	Upright cycle ergometer	Slope until respiratory compensation point	Functional capacity declines over 14 months	Sample size, single center
Banydeen et al.	2023	Prognostic value of spirometry, CPET and biomarker staging in ATTR‐CA	Retrospective, multi center	82	ATTR	Upright cycle ergometer	Unknown	Combined prognostic value of peak VO_2_, FVC and ATTR biomarker	Retrospective, small number
Bartolini et al.	2021	Response to exercise in ATTR cardiomyopathy, and correlation of VO_2_ and VE/VCO_2_ slope with clinical, biohumoral, and echocardiographic parameters.	Prospective, single center	72	ATTR	Upright cycle ergometer	Slope until respiratory compensation point	Patients showed abnormal CPET results, arterial blood pressure and heart rate responses to exercise	Sample size, single center
Bhutani et al.	2021	Correlation of CPET measurements with biomarkers and with prognosis	Retrospective, single center	41	AL and ATTR	Exercise Treadmill	Full slope	Decreased peak VO_2_ in patients with cardiac amyloidosis, Peak VO_2_ correlates with cardiac biomarkers	Alterations in biomarkers levels
Briasoulis et al.	2022	Identify clinical, biochemical, and imaging markers associated with exercise capacity impairment	Prospective, single center	23	AL	Upright cycle ergometer	Unknown	CPET results correlated with established prognostic markers	Sample size, no control group
Dalia et al.	2021	Prognostic value of CPET in patients with wild‐type transthyretin cardiac amyloidosis treated with tafamidis	Retrospective, single center	33	ATTR	Not specified	Full slope	CPET provides predictors of early worse outcomes	Sample size, single center
Hashimoto et al.	2024	Determinants of impaired exercise capacity in ATTR	Retrospective, single center	32	ATTR	Upright cycle ergometer	Unknown	Right ventricular–pulmonary artery coupling estimated by the TAPSE/PASP ratio determines exercise capacity in ATTR patients	Sample size, single center, observational, protcol not pre‐specifid
Hein et al.	2018	Predictive value of CPET parameters in patients with CA	Retrospective, single center	27	AL and ATTR	Semirecumbent cycle ergometer	Slope until respiratory compensation point	Peak VO_2_ is predictive for outcome in patients with CA	Retrospective, small number, submaximal tests
Monfort et al.	2022	Evalaute abnormal ventilatory efficiency in transthyrerin hereditary CA	Retrospective, single center	41	ATTR	Upright cycle ergometer	Slope until respiratory compensation point	High ventilatory stimulation during exercise is the main determinant of ventilatory inefficiency in ATTRv	Sample size, right ventricular measuerments
Nakaya et al.	2023	Evaluation of patients with ATTR‐CM after 1 year of tafamidis treatment	Prospective, single center	8	ATTR	Upright cycle ergometer	Slope until respiratory compensation point	Changes in exercise tolerance in patients with ATTR one year after disease specific therapy.	Small sample size, lack of control group, no cardiac rehab
Nicol et al.	2021	Functional assessment and prognosis in patients with CA assessed by CPET	Retrospective, multicenter study	150	AL and ATTR	Upright cycle ergometer	Full slope	CPET is helpful in assessing functional capacity, circulatory and chrontoropic responses as well as prognosis	Not reported
Patel et al.	2024	Characterize spectrum of functional phenotypes, association of CPET with amyloid burden and prognosis	Prospective, single center	506	ATTR	Semirecumbent cycle ergometer	Slope until respiratory compensation point	ATTR amyloidosis is characterized by distinct patterns of functional impairments between disease phenotypes	Unable to explore lung amyloid infiltration
Shibata et al.	2024	Evaluate FETCO_2_ in CA via CPET	Retrospective, single center	16	AL and ATTR	Upright cycle ergometer	Slope until respiratory compensation point	Patients with CA may find it difficult to increase cardiac output during exercise due to severe diastolic dysfunction	Retrospective, small sample size, no follow‐up
Silverii et al.	2023	Investigate cardio‐circulatory exercise response in ATTR‐CM and to evaluate the progrnostic capacity of CPET	Prospective, single‐center	75	ATTR	Upright cycle ergometer	Unknown	CPET is safe and a useful prognostic tool in patients with ATTR	Sample size, Single Center, follow‐up duration
Wernhart et al.	2024	Awheter non exertional variables of CPET provide additional information to comparison to traditional peak oxygen consumption	Retrospective, single center	21	AL and ATTR	Upright cycle ergometer	Full slope	Exertional and non‐exertional CPET performance was inferior in CA compared to HF patients	Retrospecitve, sample size, Combining AL and ATTR in one cohort
Willixhofer et al.	2024	Evaluation of predominant exercise limitations in CA versus HF	Prospective, multicenter	267	AL and ATTR	Upright cycle ergometer	Slope until respiratory compensation point	A high VE/VCO2 slope and an earlier AT was assessed in patients with CA compared to HF	CPET evaluation not centralized, difference in reporting between centers
Yunis et al.	2019	Identify CPET risk factors of mortality	Prospective, single center	56	ATTR	Exercise treadmill	Full slope	CPET testing may be used in ATTRwt to provide objective measures of prognosis and may assist in assessing outcomes.	Refferal bias possible, sample size, 30% of patients did not reach AT

*Note*: The table presents an overview of studies focused on the assessment of CPET in patients with cardiac amyloidosis. It includes details on the study year, aim, study design, sample size, diagnosis, CPET assessment methods, and the main conclusions and reported limitations of each study.

Abbreviations: AL, amyloid light chain amyloidosis; AT, anaerobic threshold; ATTR, transthyretin amyloidosis; CA, cardiac amyloidosis; CPET, cardiopulmonary exercise testing; FETCO₂, fraction of end‐tidal carbon dioxide; FVC, forced vital capacity; HF, heart failure; *N*, sample size; TAPSE/PASP, tricuspid annular plane systolic excursion to pulmonary artery systolic pressure ratio; VE/VCO₂ slope, ventilatory efficiency (ratio of minute ventilation to carbon dioxide production).

### 3.1 Participants and study characteristics

The pooled baseline characteristics for all 17 studies revealed significant heterogeneity across studies. The overall mean age was 72 years (95% CI: 69–76), predominantly male gender (85%) and left ventricular ejection fraction was 53% (95% CI: 51–54) suggest a trend towards these demographic and clinical features in the studied population. The only variable with low heterogeneity (*I* ^2^ = 0, *p* = 0.67) over four of 17 studies was serum creatinine with levels of 1.33 (1.27–1.40) mg/dL.

Most studies were single center (14 of 17) with a common limitation reported regarding small sample sizes. The investigated CPET modalities were upright cycle ergometry in 12 studies, semi‐recumbent cycle ergometry in two studies, exercise treadmill in two studies, and unspecified in one study. The discrete populations, study design, aim, and conclusions of the included studies can be seen in Table 1.

### 3.2 Reporting of CPET

Prior evaluation of reported CPET variables data for peak exercise was excluded for three studies, due to submaximal effort in two and short exercise duration (8 min) in one study (Dalia et al., 2021; Hein et al., 2018; Wernhart et al., 2024).

In the overall CA cohort, there was high heterogeneity in all variables between studies. Pooled mean values can be found in Table 2.

**TABLE 2:** Summary of CPET parameters across cardiac amyloidosis cohorts and subgroups.

Variable	Overall mean (95% CI)	*I* ^2^ (95% CI)	*p* Value of heterogenity	Studies (*n*)	Patients (*n*)
CA overall					
Peak HR, beats/min (95% CI)	118 (115–121)	0.7 (0.39–0.86)	0.001	8	1132
Peak load, Watts (95% CI)	71 (67–76)	0.72 (0.43–0.86)	<0.001	8	1086
Peak O_2_ pulse, mL/beat (95% CI)	9.1 (8.0–10.2)	0.96 (0.94–0.98)	< 0.001	5	960
Peak PETCO_2_, mmHg (95% CI)	31.0 (29.0–33.1)	0.95 (0.91–0.98)	< 0.001	4	825
Peak RER (95% CI)	1.15 (1.12–1.18)	0.95 (0.93–0.97)	< 0.001	10	1172
**Peak VE, L/min (95% CI)**	**51.1 (49.3–52.9)**	**0.57 (0–0.9)**	**0.13**	**2**	**773**
Peak VO_2_, mL/min/kg (95% CI)	14.7 (14.2–15.2)	0.67 (0.43–0.81)	<0.001	14	1416
VO_2_ at AT, mL/min/kg (95% CI)	10.1 (9.2–11.0)	0.97 (0.96–0.98)	< 0.001	11	1212
VE/VCO_2_ Slope (95% CI)	35.9 (34.1–37.7)	0.96 (0.94–0.97)	< 0.001	17	1503
AL					
Peak HR, beats/min (95% CI)	115 (108–121)	NA (NA‐NA)	NA	1	27
Peak load, Watts (95% CI)	83 (69–97)	NA (NA‐NA)	NA	1	27
Peak O_2_ pulse, mL/beat (95% CI)	9.7 (8.8–10.6)	NA (NA‐NA)	NA	1	27
**Peak PETCO**_**2**_, **mmHg (95% CI)**	**30.1 (27.6–32.6)**	**0.67 (0–0.93)**	**0.080**	**2**	**50**
**Peak RER (95% CI)**	**1.16 (1.10–1.21)**	**0.7 (0–0.93)**	**0.070**	**2**	**50**
Peak VE, L/min (95% CI)	49.6 (43.0–56.2)	NA(NA‐NA)	NA	1	23
Peak VO_2_, mL/min/kg (95% CI)	15.4 (12.8–17.9)	0.88 (0.68–0.96)	<0.001	3	67
VO_2_ at AT, mL/min/kg (95% CI)	10.8 (9.5–12.2)	NA (NA‐NA)	NA	1	27
VE/VCO_2_ slope (95% CI)	34.7 (30.9–38.5)	0.86 (0.65–0.94)	<0.001	4	82
ATTR overall					
Peak HR, beats/min (95% CI)	120 (116–123)	0.72 (0.35–0.88)	0.003	6	821
**Peak load, Watts (95% CI)**	**74 (71–76)**	**0.36 (0–0.74)**	**0.17**	**6**	**774**
Peak O_2_ pulse, mL/beat (95% CI)	9.3 (8.1–10.5)	0.84 (0.6–0.94)	<0.001	4	664
Peak PETCO_2_, mmHg (95% CI)	31.2 (29.3–33.2)	0.95 (0.89–0.98)	<0.001	3	666
Peak RER (95% CI)	1.14 (1.10–1.18)	0.95 (0.91–0.97)	< 0.001	6	798
**Peak VE, L/min (95% CI)**	**51.5 (49.1–53.8)**	**0.72 (0–0.94)**	**0.061**	**2**	**627**
**Peak VO**_**2**_, **mL/min/kg (95% CI)**	**14.5 (14.3–14.8)**	**0.28 (0–0.64)**	**0.178**	**11**	**1064**
VO_2_ at AT, mL/min/kg (95% CI)	10.2 (9.2–11.3)	0.96 (0.95–0.98)	<0.001	8	830
VE/VCO_2_ slope (95% CI)	35.3 (33.0–37.6)	0.95 (0.93–0.96)	<0.001	12	1074
ATTRwt					
Peak HR, beats/min (95% CI)	118 (110–125)	0.91 (0.68–0.97)	0.001	2	552
**Peak load, Watts (95% CI)**	**73 (70–76)**	**0.51 (0–0.86)**	**0.13**	**3**	**568**
**Peak O**_**2**_ **pulse, mL/beat (95% CI)**	**10.3 (10.0–10.5)**	**0.67 (0–0.91)**	**0.047**	**3**	**566**
Peak PETCO_2_, mmHg (95% CI)	30.6 (28.0–33.3)	0.96 (0.87–0.98)	< 0.001	2	559
**Peak RER (95% CI)**	**1.11 (1.09–1.14)**	**0.74 (0.26–0.91)**	**0.010**	**4**	**623**
Peak VE, L/min (95% CI)	52.9 (50.9–55.0)	NA (NA‐NA)	NA	1	229
**Peak VO**_**2**_, **mL/min/kg (95% CI)**	**14.5 (14.2–14.8)**	**0.02 (0–0.85)**	**0.38**	**4**	**625**
VO_2_ at AT, mL/min/kg (95% CI)	10.2 (8.6–11.8)	0.98 (0.96–0.99)	<0.001	5	636
**VE/VCO**_**2**_ **slope (95% CI)**	**38.4 (36.9–40.0)**	**0.57 (0–0.84)**	**0.052**	**5**	**656**
ATTRv					
Peak HR, beats/min (95% CI)	119 (109–128)	0.76 (0.19–0.93)	0.017	3	162
**Peak load, Watts (95% CI)**	**72 (66–77)**	**0 (NA‐NA)**	**0.60**	**2**	**121**
**Peak O**_**2**_ **pulse, mL/beat (95% CI)**	**9.8 (9.1–10.5)**	**0 (NA‐NA)**	**0.62**	**2**	**121**
Peak PETCO_2_, mmHg (95% CI)	32.5 (29.9–35.1)	0.71 (0.02–0.92)	0.031	3	162
**Peak RER (95% CI)**	**1.08 (1.06–1.10)**	**0 (NA‐NA)**	**1.00**	**2**	**121**
**Peak VE, L/min (95% CI)**	**46.1 (43.0–49.2)**	**0 (NA‐NA)**	**0.70**	**2**	**121**
Peak VO_2_, mL/min/kg (95% CI)	14.1 (12.00–16.3)	0.78 (0.29–0.93)	0.011	3	162
VO_2_ at AT, mL/min/kg (95% CI)	10.4 (8.1–12.7)	0.89 (0.71–0.96)	<0.001	3	162
**VE/VCO**_**2**_ **slope (95% CI)**	**37.9 (35.1–40.7)**	**0.7 (0–0.91)**	**0.037**	**3**	**162**

*Note*: Pooled CPET values across the overall CA cohort and subgroups, including AL amyloidosis, ATTR overall, ATTRwt, and ATTRv. Data are reported as mean (95% confidence interval) for each variable, alongside heterogeneity statistics (*I* ^2^ and *p* value). The number of studies and total patients included for each parameter are also indicated. *I* ^2^ represents the percentage of variability in effect estimates due to heterogeneity rather than chance, with higher values indicating greater heterogeneity. The *p*‐value for heterogeneity assesses the statistical significance of observed heterogeneity, with values below 0.05 suggesting significant heterogeneity. Values with nonsignificant heterogeneity are printed in bold.

Abbreviations: AL, amyloid light chain amyloidosis; AT, anaerobic threshold; ATTR, transthyretin amyloidosis; ATTRv, variant transthyretin amyloidosis; ATTRwt, wild‐type transthyretin amyloidosis; CA, cardiac amyloidosis; CI, confidence interval; CPET, cardiopulmonary exercise testing; HR, heart rate; *I* ^2^, heterogeneity index; *N*, sample size; O₂ pulse, oxygen uptake per heartbeat; PETCO₂, end‐tidal partial pressure of carbon dioxide; p, probability value; RER, respiratory exchange ratio; VE, minute ventilation; VE/VCO₂ slope, ventilatory efficiency (ratio of minute ventilation to carbon dioxide production); VO₂, oxygen uptake.

For patients with AL amyloidosis, peak end‐tidal partial pressure of carbon dioxide (PETCO_2_) and peak respiratory exchange ratio (RER) demonstrated comparability across studies. The pooled mean for peak PETCO_2_ was 30.1 mmHg (95% CI: 27.6–32.6, *I* ^2^ = 0.67, *p* = 0.080 for heterogeneity), and for peak RER, 1.16 (95% CI: 1.10–1.21, *I* ^2^ = 0.70, *p* = 0.070 for heterogeneity), both exhibiting high variability indicated by the respective *I* ^2^ values.

Within the ATTR amyloidosis cohort, consistency was noted for peak load and peak VO_2_. The pooled mean for peak load was 74 Watts (95% CI: 71–76, *I* ^2^ = 0.36, *p* = 0.17 for heterogeneity), based on six studies with moderate heterogeneity. Peak VO_2_, reported in 11 studies involving 1064 patients, had a moderately heterogenous pooled mean of 14.5 mL/min/kg (95% CI: 14.3–14.8, *I* ^2^ = 0.28, *p* = 0.18 for heterogeneity).

In the ATTRwt subgroup, high and moderate heterogeneity persists, including peak load (73 Watts, 95% CI: 70–76, *I* ^2^ = 0.51, *p* = 0.13 for heterogeneity), peak O_2_ pulse (10.3 mL/beat, 95% CI: 10.0–10.5, *I* ^2^ = 0.67, *p* = 0.047 for heterogeneity), peak VO_2_ (14.5 mL/min/kg, 95% CI: 14.2–14.8, *I* ^2^ = 0.02, *p* = 0.38 for heterogeneity), and VE/VCO_2_ slope (38.4, 95% CI: 36.9–40.0, *I* ^2^ = 0.57, *p* = 0.052 for heterogeneity).

For patients with ATTRv, comparable parameters included peak load, peak O_2_ pulse, peak RER, peak VE, and VE/VCO_2_ slope. The pooled mean for peak load was 72 Watts (95% CI: 66–77, *I* ^2^ = 0, *p* = 0.60 for heterogeneity). The pooled mean for peak O_2_ pulse was 9.8 mL/beat (95% CI: 9.1–10.5, *I* ^2^ = 0, *p* = 0.62 for heterogeneity). Peak RER was reported with a mean of 1.08 (95% CI: 1.06–1.10, *I* ^2^ = 0, *p* = 1.0 for heterogeneity), and peak VE had a pooled mean of 46.1 L/min (95% CI: 43.0–49.2, *I* ^2^ = 0, *p* = 0.70 for heterogeneity). The VE/VCO_2_ slope was reported with a pooled mean with high heterogeneity of 37.9 (95% CI: 35.1–40.7, *I* ^2^ = 0.70, *p* = 0.037 for heterogeneity). Further details on CPET variables between studies can be seen in Table 2.

### 3.3 ATTR versus AL and differences between exercise modalities

**FIGURE 2:**
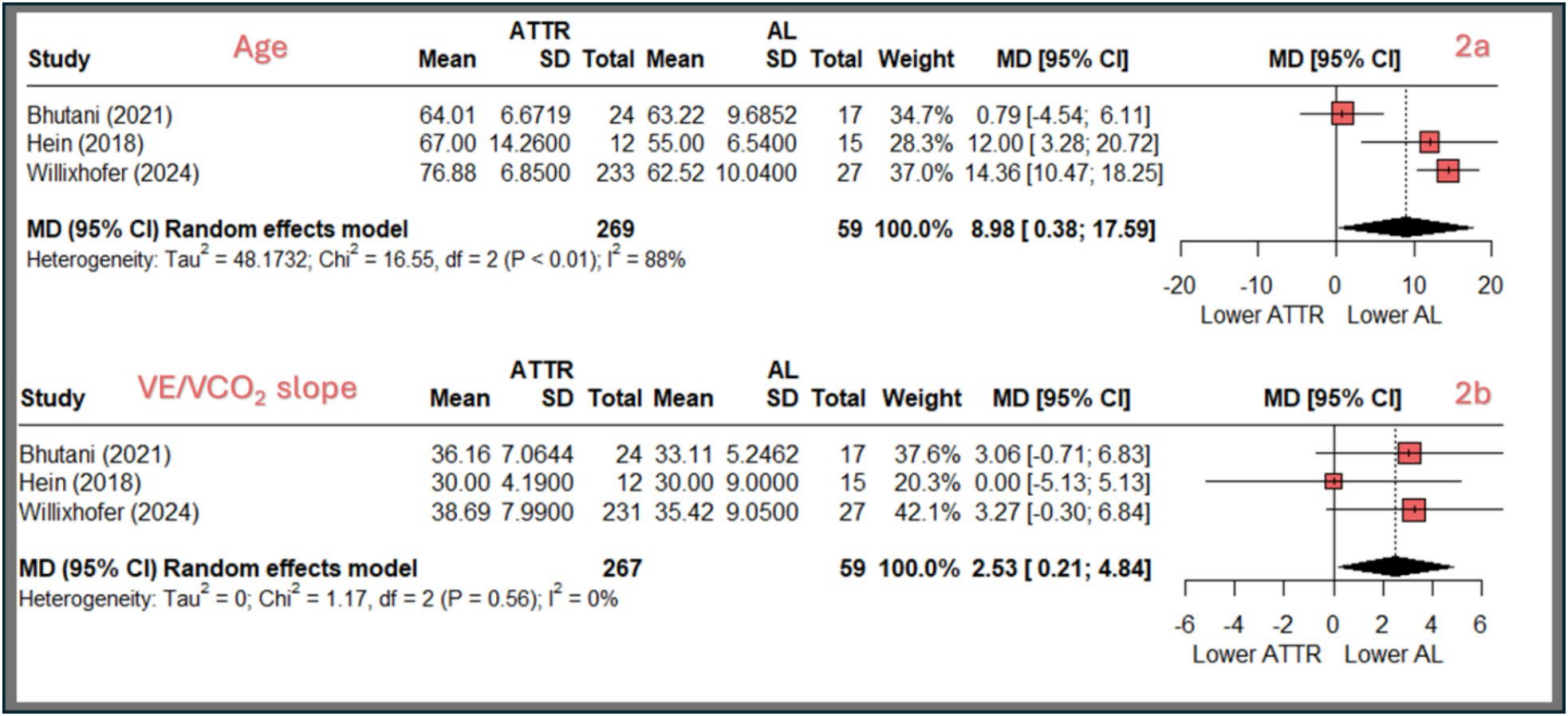
Comparative analysis of ATTR versus AL patients. Figure 2 contains two forest plots comparing patients with ATTR and AL. (a) Compares mean age, showing that ATTR patients are significantly older by 9.0 years (95% CI: 0.4–17.6, *p* = 0.04), with high variability between studies (*I* ^2^ = 87.9%). (b) Compares VE/VCO_2_ slope, indicating slightly reduced ventilatory efficiency in AL patients, with a mean difference of 2.5 (95% CI: 0.2–4.8, *p* = 0.03) and low variability (*I* ^2^ = 0%). Each study is represented by a square (mean difference) and horizontal line (confidence interval), while the diamond at the bottom shows the pooled overall estimate. AL, light chain amyloidosis; ATTR, transthyretin amyloidosis; Chi^2^, chi‐squared statistic; CI, confidence interval; df, degrees of freedom; *I* ^2^, heterogeneity index; MD, mean difference; SD, standard deviation; Tau^2^, between‐study variance; VE/VCO₂, ventilation to carbon dioxide production ratio.

The specific comparative analysis between ATTR and AL patients in a few studies (Bhutani et al., 2021; Hein et al., 2018; Willixhofer et al., 2025) revealed significant differences. Figure 2a illustrates an age difference, with ATTR patients being older by a mean of 9.0 years (95% CI: 0.4–17.6, *p* = 0.04, *I* ^2^ = 87.9%, *p* < 0.001). Figure 2b compares the VE/VCO_2_ slope between groups, showing a mean difference of 2.5 (95% CI: 0.2–4.8, *p* = 0.032, *I* ^2^ = 0%), indicating slightly reduced ventilatory efficiency in AL patients.

**FIGURE 3:**
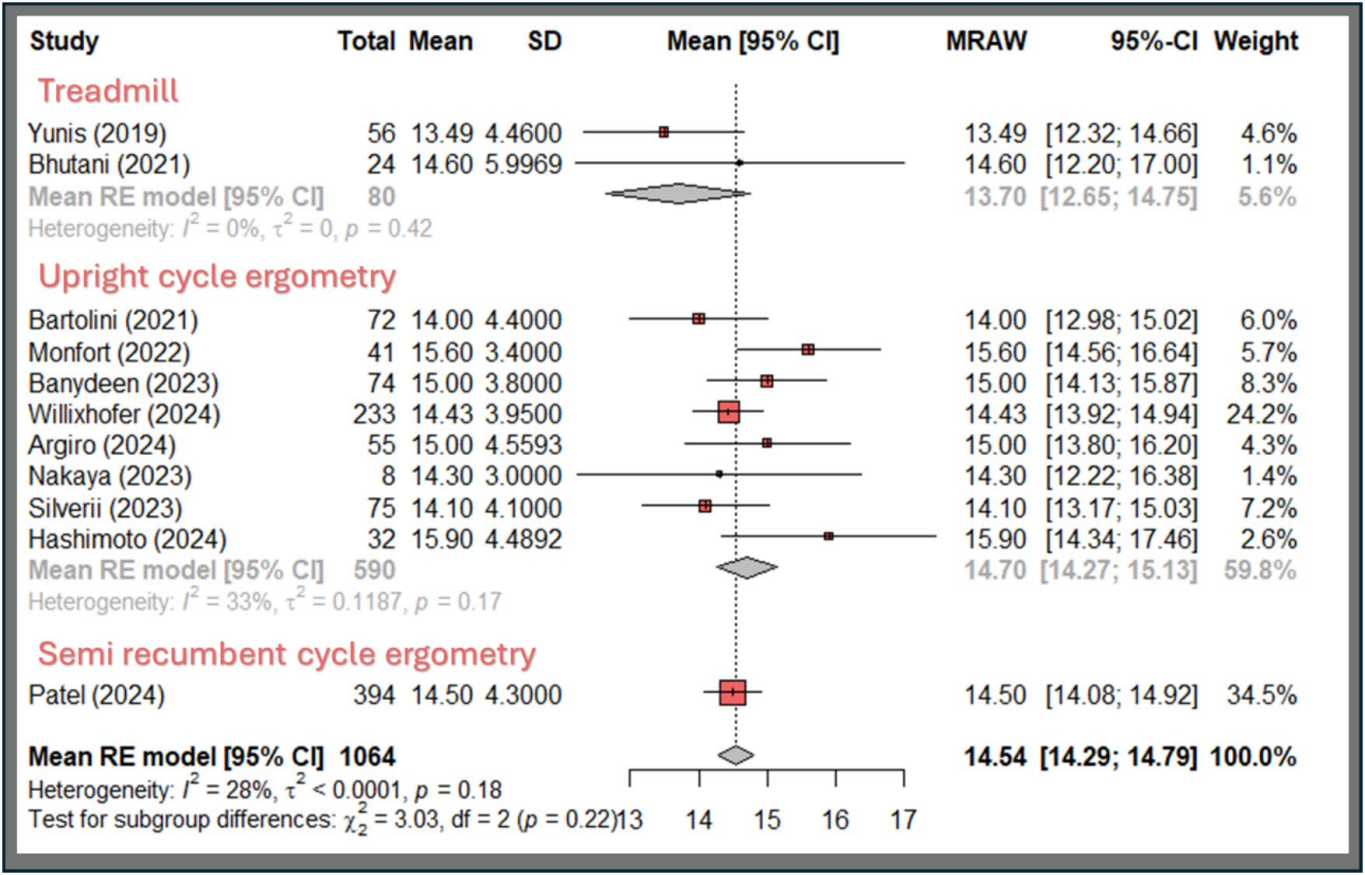
Effect of exercise modality on peak VO_2_. Figure 3 shows a forest plot, that summarizes the effect of exercise modality on peak VO_2_ during CPET, comparing treadmill, upright, and semi‐recumbent cycle ergometry. Treadmill testing yielded the lowest peak VO_2_. Each study's contribution is shown by a square (mean VO_2_) and horizontal line (confidence interval), with pooled subgroup means represented by diamonds. CI, confidence interval; *I* ^2^, heterogeneity; MRAW, mean random effects model; *p*, probability value; RE, random effects; VO₂, oxygen uptake; *τ* ^2^, tau‐squared (between‐study variance); *χ* ^2^, chi‐squared (test of heterogeneity).

The effect of exercise modality on CPET outcomes, as shown in Figure 3, highlights the potential influence of modality on peak VO_2_. In ATTR patients, treadmill testing yielded a trend for the lowest pooled peak VO_2_ at 13.7 mL/min/kg (95% CI: 12.7–14.8, I^2^ = 0%), compared to 14.7 mL/min/kg (95% CI: 14.3–15.1, I^2^ = 33%) for upright cycling and 14.5 mL/min/kg (95% CI: 14.1–14.9) for semi‐recumbent cycling. These differences suggest a trend of peak VO_2_ outcomes in relation to exercise modality, with treadmill testing producing lower values.

## 4 DISCUSSION

This systematic review and meta‐analysis represents the initial pooled evaluation of CPET parameters across CA cohorts and their respective diagnosis‐specific subgroups. Within the ATTR cohort, patients with either ATTRwt or ATTRv had an elevated VE/VCO_2_ slope, although with a considerable amount of heterogeneity between studies, indicating significant ventilatory inefficiency. Notably, when comparing ATTR and AL amyloidosis, ATTR patients exhibited a more pronounced elevation in the VE/VCO_2_ slope, potentially influenced by age and gender (Kato et al., 2018; Salvioni et al., 2020). Furthermore, exercise modality in ATTR for CPET might be of significance.

### 4.1 The high VE/VCO_2_ slope in ATTR

The overall consistently elevated VE/VCO_2_ slope in ATTRwt and ATTRv underscores the impact of disease‐specific ventilatory and hemodynamic limitations in these cohorts (Table 2) which compared to heart failure shows consistently higher values indicating worse ventilatory efficiency in general (Martens et al., 2023; Willixhofer et al., 2025).

Condensing the individual hypotheses (Argirò et al., 2024; Bartolini et al., 2021; Hashimoto et al., 2024; Monfort et al., 2022; Nicol et al., 2021; Wernhart et al., 2024; Yunis et al., 2019) and the pooled analysis for the VE/VCO_2_ slope mechanics, we hypothesize that patients with ATTR exhibit an elevated VE/VCO_2_ slope due to lung congestion, reduced ventricular compliance, right and left ventricular dysfunction, abnormalities of pulmonary perfusion, including exercise‐induced pulmonary hypertension (Holt et al., 2023), peripheral mechanisms including respiratory muscular weakness, neuropathy potentially altering mechano‐ and metaboreflexes, and autonomic dysfunction (González‐Duarte et al., 2019; Vergaro et al., 2021). Other limitations, needing consideration, include the high prevalence of pulmonary restriction, caused by amyloid deposition (shown to be up to 60%) which leads to a blunted increase of tidal volume resulting in a higher ventilatory drive during exercise (Argirò et al., 2024; Cappelli et al., 2018).

### 4.2 ATTR versus AL and differences in exercise modality in ATTR

The comparison between ATTR and AL amyloidosis (Table 2, Figure 2) potentially underscores key differences in exercise physiology. The less pronounced elevation in VE/VCO_2_ slope observed in AL compared to ATTR suggests distinct patterns of ventilatory inefficiency, comparable to previous findings (Willixhofer et al., 2025). However, age differences are likely responsible for the reported differences. Indeed, VE/VCO_2_ slope values increase in patients with HF with age and are also higher in female patients (Kato et al., 2018; Salvioni et al., 2020). Further, the heterogeneity in the overall CA group suggests an even stronger difference between exercise physiological responses. This in turn might imply to rethink the evaluation of exercise capacity and its implications for prognosis and symptomatic therapy. However, to properly evaluate the proposed hypotheses in detail, there is a need for advanced study protocols evaluating those mechanisms from rest to exercise in combination with bigger uniform sample sizes as there is a quite large gap between the number of patients with AL as compared to ATTR in CPET studies. This might be implied by the management strategy for patients, as AL is often managed by hematologists and only assessed by cardiologists for cardiac involvement of the disease with evaluations other than CPET (Kittleson et al., 2023).

The comparison of the exercise modality (Figure 3), showing a trend for worse peak VO_2_ during treadmill exercise as compared to cycle ergometry in patients with ATTR, might imply disease‐specific characteristics, which could be explained by the high prevalence of polyneuropathy in patients with both AL (17% to 35%) and ATTR (about 30% for wt and up to 80% in certain variants of the disease). In fact, peak VO_2_ during treadmill exercise has been shown to be higher as compared to cycling ergometry in patients with heart failure, potentially related to differences in muscle utilization between exercises (Mazaheri et al., 2021). This highlights potential polyneuropathic limitation in patients with ATTR further (Kittleson et al., 2023). In fact, the differences in protocols for treadmill CPET compared to cycle ergometry CPET might explain the differences partly as step protocols are often used for treadmill testing as compared to ramp protocols in cycle ergometry (Agostoni & Dumitrescu, 2019). However, for the two studies utilizing treadmill exercise, the first (Bhutani et al., 2021) used a ramp protocol, which is comparable to cycle ergometry protocols, and the second test was clearly maximum with a median RER above 1.1. Both studies, considering ramping and maximum effort, should yield higher results as cycle ergometry, analogous to HF; however, this was not the case strengthening our argument of potential polyneuropathic limitations.

### 4.3 Reference values and gender differences

The establishment of reference values for CPET variables for CA overall as well as ATTR and AL remains challenging due to variability in the parameters reported and the high heterogeneity across studies, even considering differences in CPET modality (upright bike vs. semi recumbent vs. treadmill) (Cantone et al., 2023). However, as the first meta‐analysis pooling CPET data with a reasonable sample size for a rare cardiac disease, the results with a low heterogeneity provide a cautious framework for clinical studies. The values with low heterogeneity should be validated further using large‐scale prospective observational data to ensure accuracy and applicability.

CA and especially ATTR is known to affect a predominantly male population having distinct pathophysiological mechanisms compared to their female counterparts. In fact, women have worse right ventricular and diastolic function potentially worsening ventilatory efficiency. In the pooled cohort of this meta‐analysis, 85% of patients were male, showcasing a gender‐related gap that must be addressed in future studies. At best, there is a need to establish subgroup analyses where possible. Recently, this issue has been addressed in patients with heart failure to improve prognostication via CPET. This highlights the need to evaluate gender aspects within CA to allow improved gender‐specific prognostication (Salvioni et al., 2023).

### 4.4 Diagnosis, therapy optimization, and transplant decision

Following our meta‐analysis, the VE/VCO_2_ slope emerges as a potentially valuable tool for diagnosis, disease monitoring, and therapy optimization in ATTR. From the current point of view, the VE/VCO_2_ slope might be used to additionally guide the diagnostic workup of patients with ATTR, as it might help during the early identification of cases in conjunction with standard of care imaging (e.g., echocardiography).

The VE/VCO_2_ slope is likely influenced by pulmonary congestion (De Martino & Agostoni, 2022), which could inform improved diuretic management strategies. This may include initiating earlier diuretic therapy or adopting more aggressive pulmonary interventions during clinical follow‐up, particularly given the predisposition of patients with CA to volume overload and pleural or pericardial effusions (Binder et al., 2021).

Further, CPET has been shown to refine prognosis in patients with CA, which might enable advanced prognostication comparable to the MECKI‐score initiative that serves as a well‐adapted prognostic tool for patients with heart failure (Agostoni et al., 2013; Salvioni, Bonomi, et al., 2020). Additionally, new prognostic tools might utilize the VE/VCO_2_ slope to guide transplant decisions in patients with CA. A recent review identified pleural effusions as critical red flags for heart transplantation in both AL and ATTR amyloidosis, which, when considered alongside the patient's individual VE/VCO_2_ slope, may serve as a decisive factor in transplantation decisions. Moreover, as peak VO_2_ seems to be lower in treadmill exercise, the emphasis on VE/VCO_2_ slope above peak VO_2_ for transplant decision might be justifiable (Piepoli et al., 2020; Salvioni, Bonomi, et al., 2020).

### 4.5 Limitations

Despite analyzing a sizable population for a rare cardiac disease, this systematic review and meta‐analysis has several limitations. One major limitation is the persistent heterogeneity observed across studies, even after conducting subgroup analyses. These analyses stratified data by age, CPET modality, and amyloidosis subtype (e.g., AL vs. ATTR and ATTRwt vs. ATTRv) to explore potential sources of variability. However, significant heterogeneity remained, reflecting differences in study designs, reporting practices, and patient characteristics. Further, the relatively small sample sizes necessitated the need for random effect models to adjust for skewed sample sizes, which might in turn have introduced variability and reduced estimate precision.

Additionally, to ensure reliable evaluation of peak exercise parameters, we excluded three studies: one due to short exercise duration (<8 min) and two others due to submaximal effort. While this exclusion improved data quality for peak exercise analyses.

Another limitation lies in the reliance on observational studies, as no clinical trials met the inclusion criteria. Observational designs are inherently prone to biases, including selection bias and variability in study quality. Although we used the Newcastle‐Ottawa Scale (NOS) to assess and mitigate the risk of bias, these factors may still limit the robustness of the findings.

Results in the overall CA cohort were not well comparable due to high heterogeneity among studies. This likely reflects the diverse disease phenotypes present within the studied population, emphasizing the challenges of drawing generalized conclusions about CA using CPET data. However, as disease often presents as distinct disease entities, there is a need to properly dissect diagnoses and phenotypes, which was challenging due to the study design of the selected studies, as CPET was often reported for their overall CA cohort.

Lastly, the variability in reporting CPET parameters across studies further constrained the synthesis of results. For example, some studies reported VE/VCO_2_ slope thresholds or proportions rather than raw data, precluding their inclusion in the pooled analysis. Above that, the evaluation of the VE/VCO_2_ slope was only partly reported and lacks standardization across studies, which renders a major limitation of the pooled analysis. This inconsistency highlights the need for standardized reporting to facilitate future meta‐analyses and comparisons.

### 4.6 Future directions

Future research should focus on standardizing CPET reporting to improve data comparability and enable more robust meta‐analyses. Phenotype‐specific analyses will be critical to unravel the distinct pathophysiological mechanisms underlying ATTR and AL amyloidosis. Additionally, exploring the impact of gender differences and integrating CPET data with advanced imaging and biomarker studies could enhance our understanding of disease progression and optimize patient management, including heavy treatment decisions, including transplantation and mechanical circulatory support. Large‐scale, prospective studies with uniform protocols are needed to refine these preliminary insights and establish CPET as a core tool in the evaluation and management of cardiac amyloidosis.

## 5 CONCLUSIONS

This systematic review and meta‐analysis underscores the pervasive ventilatory inefficiency observed in CA, particularly in ATTR. Differences in exercise physiology between ATTR and AL, reflected by differences in VE/VCO_2_ slope, suggest distinct mechanisms that warrant further study. Lastly, exercise modality might influence peak VO_2_, with treadmill exercise potentially interfering with disease mechanisms.

## AUTHOR CONTRIBUTIONS

All authors meet the ICMJE authorship criteria, RW was involved in conceptualization, systematic review, and manuscript draft. ES was involved in systematic review and manuscript draft. NC was involved in statistical analysis. MC and JC were involved in manuscript draft. PA was involved in conceptualization and manuscript draft.

## FUNDING INFORMATION

This work was supported by a Career Development Fellowship by the Fondazione IEO‐MONZINO and by the Italian Ministry of Health (ricercar Corrente, CUP = B43C24000090001).

## CONFLICT OF INTEREST STATEMENT

The authors declare no conflict of interest.

## ETHICS STATEMENT

Data was derived from peer‐reviewed published original investigations, therefore no additional ethical approval was obtained.

## Supporting information


Table S1.

Appendix S1.

## Data Availability

Data will be available upon reasonable request.
