# Supplementary material for: Ventilatory efficiency in cardiac amyloidosis—A systematic review and meta‐analysis
Source: Physiol Rep. 2025 May 1;13(9):e70308. doi: 10.14814/phy2.70308 (PMC12045699; doi:10.14814/phy2.70308)
Supplement: Supplementary file 1 — Table S1. [file PHY2-13-e70308-s002.docx]

Supplemental Table 1: Bias Scoring according to the Newcastle-Ottawa Scale

Supplemental Table 1: Bias Scoring according to the Newcastle-Ottawa Scale. Each column represents a criterion assessed by the Newcastle-Ottawa Scale for evaluating study quality, with the scores ranging from 0 (not meeting the criterion) to 1 (criterion met). The rows correspond to individual studies, identified by the first author and year of publication. The total score, representing the sum of all criteria met (maximum score = 9), is shown in the last column. Highlighted columns indicate key criteria: yellow represents selection bias (columns 1-4), red represents comparability (column 5), and blue represents outcome/exposure assessment (columns 6-8). A score of 2 in the red column indicates that the study adequately controlled for its primary confounder (e.g., age, gender, or a critical variable) as well as additional confounders.
